# Socioeconomic determinants of malaria in Ugandan children: An interpretable machine learning approach for public health policy

**DOI:** 10.1371/journal.pdig.0001152

**Published:** 2026-09-03

**Authors:** Cleiane Gonçalves Oliveira, Marcos Flávio S. V. D’Angelo, Matheus P. Libório, Marcelo Perim Baldo, Hasheem Mannan

**Affiliations:** 1 Federal Institute of the North of Minas Gerais - Campus Januária, Januária, Minas Gerais, Brazil; 2 Graduate Program in Health Sciences, UNIMONTES, Montes Claros, Minas Gerais, Brazil; 3 Department of Computer Science, UNIMONTES, Montes Claros, Minas Gerais, Brazil; 4 Graduate Program in Computational Modeling and Systems, UNIMONTES, Montes Claros, Minas Gerais, Brazil; 5 Department of Pathophysiology, UNIMONTES, Montes Claros, Minas Gerais, Brazil; 6 School of Nursing, Midwifery and Health Systems, University College Dublin, Dublin, Ireland; University College London, UNITED KINGDOM OF GREAT BRITAIN AND NORTHERN IRELAND

## Abstract

Malaria remains a critical global health crisis, placing a disproportionate burden on children under five in Uganda. To transition from broad surveillance to targeted intervention, this study applies interpretable machine learning to identify key socioeconomic predictors of malaria using the 2018–2019 Uganda Malaria Indicator Survey. By employing Random Forests for feature selection and Decision Trees for classification, we addressed the inherent class imbalance using robust metrics such as the F2-score, Matthews Correlation Coefficient, and Precision-Recall Curve. Specifically, the Random Under-Sampling technique enabled the model to achieve a Recall of 77%, prioritizing the reliable detection of true positives over simple accuracy. The analysis highlights the hierarchical importance of determinants such as household size, mosquito net ownership, and maternal education. The study’s defining contribution is the extraction of explicit “if-then” rules that visualize how these factors combine to create risk profiles, particularly revealing distinct disparities across regions such as Busoga and West Nile. These interpretable findings empower policymakers with actionable, evidence-based insights, moving beyond simple prediction to facilitate the design of structural and region-specific public health strategies.

## Author summary

Malaria remains a devastating disease, especially for young children in Uganda. While we know that medical treatments and bed nets are effective, deciding exactly where and to whom these limited resources should be sent is a major challenge for public health workers. In our study, we wanted to see if we could predict a child’s risk of getting malaria just by looking at their everyday living conditions, without needing a medical test. We used a computer model to analyze survey data from thousands of Ugandan families, focusing on social factors like housing materials, family size, and the mother’s education level. Instead of creating a complex, hidden formula, our approach generated simple “if-then” rules that anyone can understand. For example, we found that even if a house has a good quality roof, a child is still at high risk if the family is large and lacks enough mosquito nets. By identifying these specific combinations of social and economic disadvantages, our findings provide a practical guide. Health authorities can use these clear rules to find the most vulnerable communities and deliver targeted help, ultimately saving lives and optimizing scarce resources.

## Introduction

Malaria continues to be one of the leading causes of mortality and morbidity worldwide, representing a significant global health challenge, particularly in sub-Saharan Africa. In 2022, approximately 249 million malaria cases were registered globally, with the African continent accounting for 95.4% of worldwide deaths, and nearly 78.1% of these deaths occurring in children under five years of age. Uganda is prominently featured among African countries, accounting for almost half of all global malaria cases, with malaria prevalence among children under 5 years old estimated at 18.2% in 2019 [1].

Public authorities in Uganda have implemented various interventions, including the distribution of long-lasting insecticide-treated bed nets and employing indoor residual spraying [2]. However, these efforts face significant bottlenecks, including the costs of rapid diagnostic tests, the need for trained personnel to administer them, and the persistent challenge of making these resources available in rural and remote areas where a significant portion of the population resides. In this context, there is a clear and urgent need for innovative and practical tools to enhance diagnosis and prevention strategies [3].

The field of Machine Learning has emerged as a powerful approach for decision-making and predictions, effectively leveraging the vast amounts of data produced by the healthcare sector [4]. ML tools offer the potential for effective, non-invasive, and often more cost-efficient methods for disease prediction, which are crucial in resource-constrained settings [5,6]. ML techniques have been widely applied across various health contexts for predicting diseases such as heart disease, diabetes, and cancer [7], and their utility extends to malaria management by assisting in diagnosis and predicting disease presence based on patient symptoms and signs [3].

Numerous studies have explored diverse machine learning approaches and algorithms for malaria prediction. For instance, Mariki et al. [3] compared the efficiency of algorithms such as Logistic Regression, K-Nearest Neighbors, Support Vector Machine, Decision Tree, and Random Forest for malaria diagnosis using clinical symptoms and patient features. Other research, such as that by Barboza et al. [8], applies deep learning models, including Long Short-Term Memory and Gated Recurrent Unit, for malaria prediction in specific geographical clusters, often integrating time-series analysis. Bayesian belief networks have also been employed to model the prediction and ranking of risk factors for malaria infections in children under five [9,10]. Alexandrov et al. [11] explored techniques such as Neural Networks, Evolutionary Algorithms, and Fuzzy Logic for developing discrete-logical decision-making systems in medical information systems, adaptable for disease prediction. More recently, studies have focused on using ensemble learning and explainable AI to enhance accuracy and interpretability in malaria diagnosis [12].

A particularly explored approach is the use of sociodemographic data for disease prediction [3,9,10]. Such data allow for the identification of external factors, beyond clinical symptoms, that favor disease onset and transmission [8]. These factors include housing materials, access to sanitation and water sources, household wealth, and maternal education level, which are consistently identified as key determinants of malaria risk [10,13]. This information is critical for identifying key factors consistently associated with disease emergence, facilitating the definition of public policies for prevention and outbreak response [3]. Understanding the complex interplay of socioeconomic factors is paramount for designing targeted and effective public health interventions [1].

Decision Tree models, due to their inherent interpretability, can be particularly valuable in this context [3,14]. They offer clear insights into the hierarchical importance of different socioeconomic features, translating complex relationships into actionable “if-then” rules that health officials can readily use [5,15]. This transparency is crucial in high-risk domains like healthcare, where “black box” methods are often unacceptable, and the rationale behind predictions must be understandable to foster trust among stakeholders. When combined with data balancing techniques, these models can effectively address the imbalanced nature of health datasets - where positive cases might be rare - ensuring robust and reliable predictions without overestimating performance [3,12].

Consequently, this work aims to go beyond simple classification accuracy. The primary objective is to leverage interpretable machine learning, specifically Decision Tree models, to extract explicit, actionable ‘if-then’ rules from socioeconomic data. By applying data-balancing techniques and prioritizing rigorous evaluation metrics such as the F2-score and Matthews Correlation Coefficient (MCC), this study seeks to identify and rank the critical non-clinical determinants of malaria—such as housing structure, regional disparities, and maternal education. This approach is designed to provide public health officials with a transparent decision-support tool that translates complex demographic data into clear, evidence-based strategies for targeted intervention and efficient resource allocation.

The remainder of this paper is organized as follows. The “Methodology” section details the data selection, preprocessing steps, statistical analysis, and the selection and evaluation of machine learning algorithms. The “Results and Discussion” section presents the findings from the algorithm performance evaluations and provides an interpretation of the results. Finally, the “Conclusion” sections discuss its implications and outlines avenues for future research.

## Materials and methods

This section outlines the methodology used to identify socioeconomic predictors of malaria in Ugandan children under 5 years of age. It covers data acquisition and selection, attribute statistical analysis, data preprocessing steps, feature selection strategy, comparison of machine learning algorithms, and specific analysis involving decision trees with balancing techniques for rule generation. All tests in this study used stratified 10-fold cross-validation.

### Data acquisition

The data utilized in this study were obtained from the 2018–2019 Uganda Malaria Indicator Survey (MIS) database. The MIS is a component of the Demographic and Health Surveys program, providing high-quality, large-scale survey data to enhance global understanding of health trends, though its cross-sectional nature and infrequent frequency can limit temporal analysis. The Ugandan database contains comprehensive sociodemographic details, household characteristics, and specific health indicators for 45,767 individuals, including 7,780 children under five with malaria test results and anemia data [2].

### Data pre-processing and increased dimensionality

The raw data from the MIS database underwent several pre-processing steps to prepare it for machine learning analysis. These steps included handling missing values and encoding nominal features to conform to Scikit-learn [16]. Categorical attributes, such as “Region of residence,” “Principal source of water for drinking,” and “Type of sanitary installation,” were transformed into numerical representations using one-hot encoding. This process, while necessary for machine learning algorithms, increased the dataset’s dimensionality. For example, a single categorical column representing “Region of residence” with 15 distinct categories was expanded into 15 binary columns.

The transformation resulted in a significant expansion of the feature set, as detailed in S1 Table in the Supporting Information, which outlines the attributes, their initial and final column counts after pre-processing. The initial dataset of 54 features was expanded to 93 independent variables and one dependent variable.

### Feature selection using Random Forest

To mitigate the effects of increased dimensionality and to identify the most impactful socioeconomic factors, feature selection was performed. The Random Forest algorithm, known for its ability to generate intrinsic variable importance metrics, was employed to rank attributes based on decreases in mean impurity or variance. This approach provides a robust basis for identifying the most influential features in malaria prediction [12,17–19].

To mitigate the well-known bias of Mean Decrease in Impurity toward high-cardinality features, our pre-processing step transformed all categorical variables using one-hot encoding. Consequently, the Random Forest algorithm evaluated almost exclusively binary features, rendering the importance estimation much more robust for this initial dimensionality reduction step.

Random Forest, with the configuration from S2 Table in the Supporting Information, was applied to the set of 93 data points. The final importance value for each attribute is the average value obtained across the cross-validation iterations. A study criterion of an importance threshold of 0.010 (at least 1%) was defined, resulting in 37 most important attributes.

### Comparison of machine learning algorithms

To demonstrate the potential of socioeconomic data for predicting malaria among Ugandan children, a comprehensive comparative analysis of machine learning algorithms was undertaken using the variables identified in the previous section.

The machine learning algorithms selected for this comparative analysis spanned various categories, including linear models (Logistic Regression, Stochastic Gradient Descent, Support Vector Classifier), tree and ensemble models (Decision Tree, Random Forest, Gradient Boosting), neural networks (Multi-layer Perceptron), distance/neighbor-based algorithms (K-Nearest Neighbors), and probability/Bayesian-based models (Gaussian Naive Bayes). The implementation was developed in Python, utilizing the Scikit-Learn library [16].

The parameters used corresponded to the common values of the respective libraries, as detailed S2 Table in the Supporting Information. This comprehensive presentation ensures transparency and facilitates the reproducibility of our methodological approach. All tests were conducted using 10-fold cross-validation, a common practice for robust model evaluation and widely recommended in literature [3,20–22]. Data were stratified to ensure that the proportions of classes in the training and testing sets mirrored those of the original dataset. Furthermore, data normalization was applied exclusively to the training set, and the same normalization model was then applied to the test set to prevent data leakage and ensure reliable results.

To thoroughly assess machine learning model performance, especially with imbalanced datasets, a broad range of metrics is crucial. Therefore, researchers commonly use metrics such as accuracy, precision, recall, F1-score, and the Area Under the Receiver Operating Characteristic Curve (AUC-ROC). Recall, which indicates the proportion of actual positive cases correctly identified, is often prioritized in malaria prediction because accurately detecting true positives is vital for effective public health interventions and disease control [3]. However, a balance between recall and precision is necessary to avoid excessive false positives that could overburden healthcare systems. The F1-score offers a balanced perspective on model performance, particularly in imbalanced datasets, by calculating the harmonic mean of precision and recall. The AUC-ROC provides a comprehensive evaluation of a classifier’s ability to differentiate between classes across various threshold settings.

Beyond these standard metrics, the F2-score, MCC, and Area Under the Curve Precision-Recall (AUC-PRC) are particularly valuable for imbalanced medical datasets. The F2-score, similar to the F1-score, is a harmonic mean of precision and recall, but it places a greater emphasis on recall. This makes it highly relevant for conditions like malaria prediction, where minimizing false negatives (missing actual cases) is often more critical than minimizing false positives. The F2-score will be the main target of this study [3].

To complement, the MCC is another robust metric for evaluating binary classifications, especially with imbalanced datasets. MCC considers all four values from the confusion matrix and produces a score between -1 and +1, where +1 signifies a perfect prediction, 0 indicates a random prediction, and -1 represents an inverse prediction. Unlike accuracy or F1-score, MCC offers a balanced measure even when class sizes differ significantly, making it a more informative and reliable metric for assessing the quality of classification models in medical diagnoses [23]. The formulas for these metrics, including F2-score and MCC, are presented in Table 1.

**Table 1:** Assessment metrics formulas.

Metric	Formula
Accuracy	(*TP* + *TN*) / (*TP* + *TN* + *FP* + *FN*)
Precision	*TP* / (*TP* + *FP*)
Recall	*TP* / (*TP* + *FN*)
F1-Score	2*(Precision*Recall)/(Precision+Recall)
F2-Score	5*(Precision*Recall)/((4*Precision)+Recall)
MCC	$(TP*TN-FP*FN)/\sqrt{\left((TP+FP)*(TP+FN)*(TN+FP)*(TN+FN)\right)}$

The AUC-PRC visualizes the trade-off between precision and sensitivity across the entire range of decision thresholds, illustrating the model’s behavior beyond a single operating point. AUC-PR is a robust, threshold-independent metric that aggregates the predictive power of the model across all potential cutoff points. Unlike static point estimates such as F-scores or the MCC, the AUC-PR captures the global stability of the classifier, providing the necessary context to select a threshold that best aligns with the specific goals of public health interventions [12].

### Decision Tree analysis

Using the features identified through the Random Forest selection process, a Decision Tree model was developed not only to assess their predictive power but also to uncover the hierarchical relationships and conditional dependencies among these socioeconomic factors for classifying malaria cases.

Hyperparameter optimization was performed to adapt the model to unbalanced data. The GridSearch technique was applied considering the parameters and values presented in the Table 2.

**Table 2:** Assessment metrics formulas.

Parameter	Values
Max_depth	3,5,8,10
Min_samples_leaf	5,10,20,50
Class_weight	Balanced, None
Criterion	Gini, Entropy

Hyperparameter optimization addressed four key aspects. The min_samples_leaf parameter was tuned to ensure sufficient sample size in terminal nodes, yielding smoother probability estimates and reducing model variance. Class imbalance was mitigated using the class_weight parameter, which assigns higher penalties to misclassifications of the minority class. Model complexity was constrained via max_depth to prevent overfitting. Finally, the splitting criterion was evaluated to determine whether Entropy (Information Gain) provided superior class separation compared to the Gini Impurity index. The average_precision metric was defined as target scoring because it does not use the standard 0.5 threshold but instead the model’s probability.

Next, the threshold that maximized the model’s recall was determined. Validation measures use a threshold of 0.5 to define, in this case study, who is or is not sick. Changing this threshold can improve the model’s performance and efficiency.

A significant challenge in health-related machine learning applications, particularly in disease prediction, is the inherent imbalance in datasets, where positive cases are often rare compared to negative cases. This imbalance can lead to models that perform poorly on the minority class, even when achieving high overall accuracy. Given the identified class imbalance in the dataset, data-balancing techniques were applied to address it. In each cross-validation iteration, the chosen balancing technique was used exclusively on the training dataset. The model was then trained on the balanced training data and evaluated on the original, unbalanced test dataset. This approach ensures that the performance assessment reflects a realistic application scenario, preventing overestimation of model performance [6].

Two oversampling and two undersampling techniques from the imbalance-learn library [24] were employed and evaluated to assess their impact on model performance for malaria prediction.

From Oversampling, Random Over-Sampling (ROS) was used, which generates additional instances of the minority class by randomly sampling with replacement to align its size with that of the majority class [22]. The second was SMOTE, which creates synthetic samples for the minority class by interpolating between existing minority-class instances, thereby expanding decision boundaries and enhancing generalization [25].

Undersampling methods were used, including Random Under-Sampling (RUS), which randomly removes instances from the majority class to balance the dataset, and Cluster Centroids, which replaces majority-class samples with the centroids of k-means clusters. And the second was Near Miss (NM), which aims to selectively remove the majority class samples based on their proximity to minority class examples. The goal is to retain the most informative instances of the majority class while reducing the dataset size.

### Generation of rules from the Decision Tree

After training the optimized Decision Tree model, a set of “if-then” rules was extracted. The interpretability of these rules will allow public health officials to gain direct insights into the relationships between various socioeconomic determinants and malaria risk, facilitating the design of targeted and effective interventions and public policies.

## Results and discussion

### Feature selection using Random Forest

The feature importance analysis, derived from applying the Random Forest model across 93 features, is detailed in Table 3. This table quantifies the relative importance of various socioeconomic factors in predicting malaria incidence by their contribution to the model’s predictive accuracy.

**Table 3:** Relative importance of features.

No.	Importance	Meaning
**1**	**0.075**	Total number of household members
**2**	**0.052**	Number of mosquito nets the household owns
**3**	**0.047**	Number of resident children under 5 years old
**4**	**0.046**	Number of rooms used for sleeping
**5**	**0.035**	Mother’s highest educational level
**6**	**0.027**	Number of children sleeping under a mosquito net last night
**7**	**0.022**	If children sleep under a mosquito net
**8**	**0.022**	If the household shares a bathroom with other households
**9**	**0.021**	Number of animals the household owns
**10**	**0.021**	If someone in the household has a bicycle
**11**	**0.021**	If has a cell phone
**12**	**0.020**	Has a chair
**13**	**0.019**	Has a radio
**14**	**0.019**	Has a table
**15**	**0.019**	If owns land suitable for agriculture
**16**	**0.018**	Main roof material from group (‘Concrete’, ‘Tiles’, ‘Asbestos’, ‘Roofing shingles’, ‘Iron sheets’)
**17**	**0.018**	Household structure relationship:two opposite-sex adults
**18**	**0.018**	Relationship to the head of the household: Son/daughter
**19**	**0.017**	Main roof material from group (‘Thatch/palm leaf’, ‘Rustic mat’, ‘Mud’, ‘Cardboard’, ‘Tarpaulin’, ‘No roof’)
**20**	**0.017**	Household structure relationship: Three or more related adults
**21**	**0.017**	Has a bed
**22**	**0.016**	Region of residence is Karamoja
**23**	**0.016**	Relationship to the head of the household: Grandchild
**24**	**0.016**	Region of residence is Busoga
**25**	**0.015**	Main source of drinking water from group (‘Piped into dwelling’, ‘Piped to yard/plot’, ‘Public tap/standpipe’, ‘Tube well or borehole’, ‘Protected well’, ‘Protected spring’, ‘Rainwater’, ‘Bottled water’, ‘Sachet water’)
**26**	**0.015**	Main source of drinking water from group (‘Unprotected well’, ‘Unprotected spring’, ‘River/dam/lake/ponds/stream/canal/irrigation channel’)
**27**	**0.015**	If has electricity
**28**	**0.014**	Region of residence is West Nile
**29**	**0.013**	Someone in the household has a bank account
**30**	**0.012**	Type of sanitary facility from group (‘Flush to piped sewer system’, ‘Flush to septic tank’, ‘Flush to pit latrine’, ‘Ventilated Improved Pit latrine (VIP)’, ‘Pit latrine with slab’, ‘Composting toilet’)
**31**	**0.012**	Type of sanitary facility from group (‘Pit latrine without slab/open pit’, ‘Hanging toilet/latrine’, ‘No facility/bush/field’, ‘Other’)
**32**	**0.011**	If the residence is in a rural area
**33**	**0.011**	Household structure relationship: one adult
**34**	**0.011**	If has a clock/watch
**35**	**0.010**	Dwelling sprayed against mosquitoes in the last 12 months
**36**	**0.010**	Region of residence is Lango
**37**	**0.010**	Region of residence is Acholi

The analysis validates the hypothesis that malaria is socially determined and presents a clear hierarchy of risk factors. A predominance of demographic and overcrowding determinants is observed. Notably, “Total number of family members” (0.075) and “Number of resident children under 5 years old” (0.047) are among the main predictors. This, combined with the importance of “Number of mosquito nets the family owns” (0.052) and “Number of bedrooms used for sleeping” (0.046), suggests that housing conditions with high population density (overcrowding) without proportional protection are the main drivers of transmission. Furthermore, the relevance of “Higher educational level of the mother” (0.035) highlights that structural interventions must be complemented by educational initiatives to be fully effective. Regional differences were also captured, with the Karamoja (0.016) and Busoga (0.016) regions appearing as important variables, reinforcing the need for geographically targeted strategies.

### Comparison of machine learning algorithms

Table 4 presents the performance metrics for various machine learning algorithms applied to the malaria prediction task.

**Table 4:** Performance comparison of machine learning algorithms.

	Accuracy	Precision	Recall	F1-score	F2-score	AUC-ROC	MCC	AUC-PR
LogisticRegression	**89**	**62**	2	4	3	77	10	30
SGDClassifier	88	46	3	5	3	76	7	30
SVM	82	19	18	18	18	60	8	17
DecisionTreeClassifier	85	31	28	29	29	62	21	18
RandomForestClassifier	87	41	24	30	26	74	**25**	31
GradientBoostingClassifier	**89**	47	6	11	7	**78**	14	**32**
MLPClassifier	88	42	20	27	22	75	23	29
KNeighborsClassifier	87	33	18	23	19	67	17	20
GaussianNB	77	26	**54**	**35**	**44**	75	**25**	28

Although models such as Logistic Regression and Gradient Boosting achieved high accuracy (89%), a deeper analysis reveals the “accuracy paradox,” common in unbalanced medical datasets. These models exhibited a critically low Recall (2% and 6%, respectively), effectively failing to identify the vast majority of real malaria cases. In a public health context, this creates a dangerous false sense of security.

The lack of significant recall improvement across these complex baseline models empirically demonstrates that algorithmic complexity alone (e.g., using Neural Networks or Gradient Boosting) cannot resolve the severe class imbalance in health data without proper balancing interventions.

On the other hand, GaussianNB achieved the highest Recall (54%) among the base models. Still, its low Precision (26%) would result in an excessive number of false positives, potentially overwhelming the healthcare system. Its interpretability can be considered weaker or less intuitive than that of rule-based models (such as Decision Trees), as the classification does not derive from a sequential, traceable logical structure of conditions, but rather from the abstract calculation of conditional probability densities.

While post-hoc explainability tools like SHAP are excellent for interpreting black-box models, we prioritized the inherent interpretability of a single Decision Tree. This choice allows for the extraction of standalone, physical ‘if-then’ rules that field workers can deploy ‘offline’ in resource-constrained settings without requiring a computational inference engine.

The Decision Tree Classifier, with moderate performance in the default settings (28% Recall and 21% MCC), emerged as the ideal candidate not only because of the numbers, but crucially because of its inherent interpretability. Unlike “black box” methods, Decision Trees allow tracing the logic behind each prediction, a prerequisite for translating statistical findings into reliable public health interventions.

**Fig 1:**
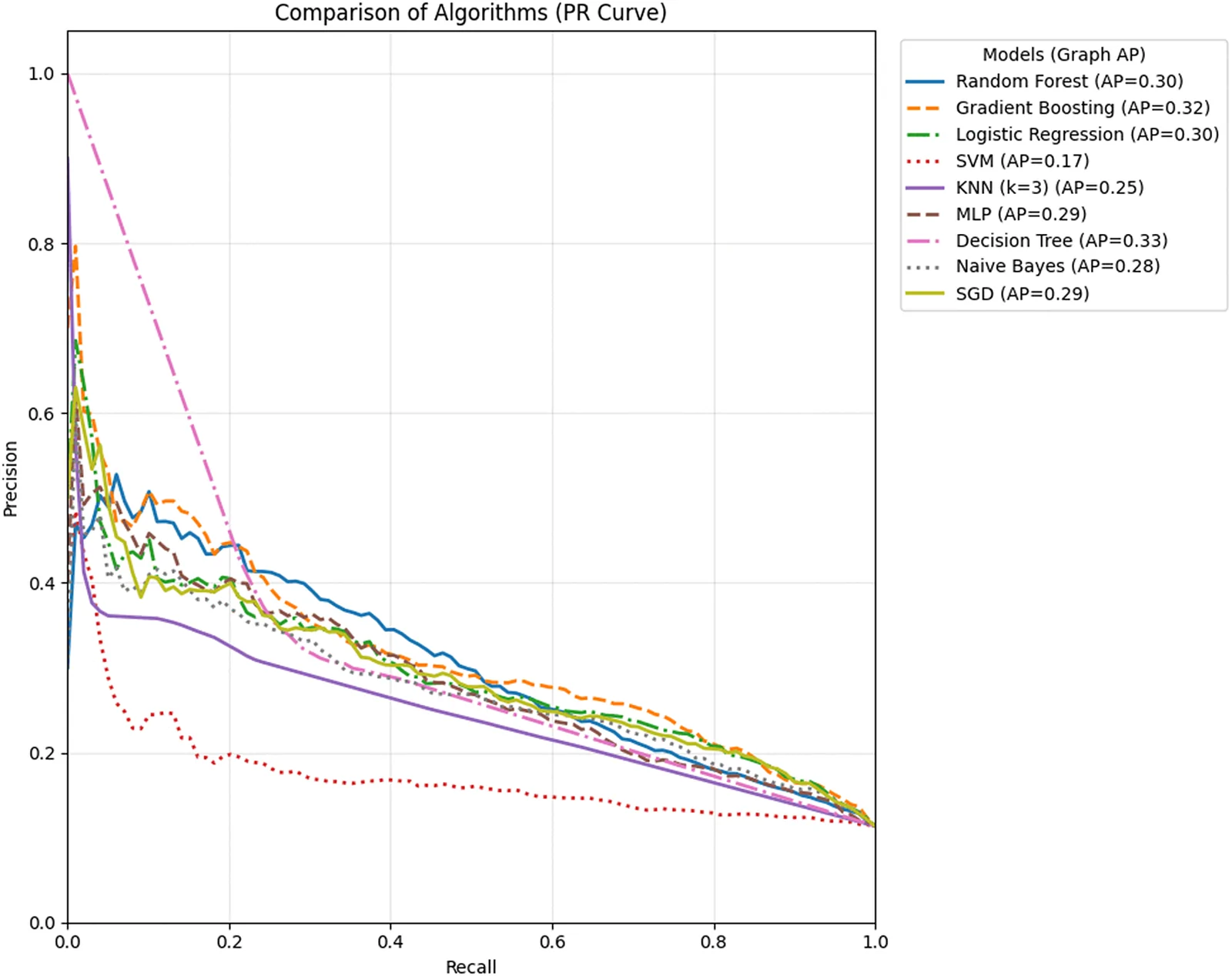
Comparison of machine learning algorithms in a Precision-Recall Curve.

Fig 1 illustrates the AUC-PR, highlighting that, although Gradient Boosting has the largest area under the mean curve (AUC-PR = 0.32), the Decision Tree (AP = 0.33) maintains a competitive balance, justifying its selection for subsequent optimization. The slight discrepancy observed between the AUC-PR values in the table and the AP in the graph legend is attributed to the order of mathematical operations during cross-validation. The value displayed in the table represents the arithmetic mean of the scores calculated individually for each fold (Mean Average Precision). In contrast, the value in the graph corresponds to the area under the mean curve (Area Under the Mean Curve), which is constructed via linear interpolation of precision and recall points across all folds to create a unified visualization. This interpolation and smoothing process introduces slight numerical approximations that naturally differ from the direct arithmetic mean.

In summary, the baseline comparison in Table 4 served strictly to validate the dataset’s predictive capacity before advancing the pipeline. While complex models like Gradient Boosting suffered from the accuracy paradox, they confirmed that algorithmic complexity alone cannot overcome class imbalance. Consequently, instead of expanding on these black-box baselines, the subsequent stages of this study focused on optimizing a Decision Tree architecture.

### Decision Tree analysis

Once the Decision Tree was selected due to the ease of rule generation, the hyperparameters were optimized (max_depth = 8, min_samples_leaf = 20, class_weight = ’balanced’, criterion = ’entropy’). Table 5 shows the evolution of the model through three stages. Decision Tree 1 (default parameters) showed a Recall of only 36% and an *F*2-score of 35%. With hyperparameter optimization, Decision Tree 2 showed a substantial improvement, raising the Recall to 67% and the *F*2-score to 47%, demonstrating the effectiveness of the class_weight parameter in penalizing the error in the minority class. To further refine the model’s sensitivity, the decision threshold was set to 0.4517, yielding Decision Tree 3. This final configuration achieved a Recall of 71% and an *F*2-score of 48%, slightly sacrificing accuracy to ensure that more infected children were correctly identified.

**Table 5:** Performance comparison of different Decision Trees.

	Accuracy	Precision	Recall	F1-score	F2-score	AUC-ROC	MCC	AUC-PR
Decision Tree 1	**83**	**31**	36	**33**	35	63	24	18
Decision Tree 2	68	21	67	32	47	**73**	**34**	**26**
Decision Tree 3	66	21	**71**	32	**48**	**73**	23	**26**

Considering Decision Tree 3 as the base model, data balancing techniques were applied to determine whether performance could be improved. The results are detailed in Table 6. The Random Under-Sampling (RUS) technique proved to be the most effective for the objective of this study. The model trained with RUS achieved the highest Recall (77%) and the highest F2-score (49%) among all variations tested.

**Table 6:** Performance comparison of different balancing techniques.

	Accuracy	Precision	Recall	F1-score	F2-score	AUC-ROC	MCC	AUC-PR
Decision Tree 3	66	21	71	**32**	48	**73**	23	**26**
RUS	61	20	**77**	**32**	**49**	**73**	23	23
NM	38	12	68	20	35	50	2	11
ROS	66	21	71	**32**	48	72	**24**	**26**
SMOTE	**75**	**23**	49	31	39	71	21	23

Although the overall accuracy dropped to 61%, this trade-off is ethically and practically acceptable in public health. From a public health perspective, the cost of misclassification is highly asymmetric. A False Negative (failing to identify a child with malaria) carries a severe risk of morbidity, mortality, and the perpetuation of the transmission reservoir. Conversely, a False Positive (classifying a healthy child as high-risk) incurs primarily an economic cost—specifically, the cost of a confirmatory Rapid Diagnostic Test.

In contrast, techniques such as NM severely degraded the model (MCC of 0.02), making it practically random. SMOTE and ROS showed inferior results to RUS in terms of Recall (49% and 71%, respectively), confirming that, for this specific dataset and for the hierarchical structure of the decision rules, the simplification of the majority class via random subsampling (RUS) allowed a better definition of the decision boundaries for the risk class. Consequently, the Decision Tree optimized with the RUS technique was used to generate the final “if-then” rules.

Therefore, the proposed Decision Tree model serves as an effective first-line triage mechanism rather than a standalone diagnostic tool. By filtering the population and identifying the risk profiles that capture nearly 80% of infections, policymakers can direct limited testing resources and bed net distribution campaigns to these specific high-risk groups, rather than relying on inefficient random screening. The model narrows the search space for health workers, ensuring that the majority of infected children are flagged for intervention, even if it requires testing some non-infected children.

### Generation of rules from the Decision Tree

The decision tree analysis originally generated 58 rules. However, to guarantee the interpretability and practical application of these findings for public health officials, we applied specific selection criteria. We exclusively chose rules that predicted the target class (high malaria risk) and that were at most 6 levels deep. This depth restriction resulted in the 10 representative rules (Rules 1–10) presented here, ensuring that the actionable ‘if-then’ conditions remain cognitively accessible while effectively illustrating the critical patterns of vulnerability.

Table 7 summarizes the ten representative rules extracted from the Decision Tree that classify a child as positive for malaria. Regional, structural, and socioeconomic determinants categorize the rules. Notably, ‘Roof Material’ and ‘Mosquito Net Ownership’ appear as recurrent discriminators across multiple profiles.

**Table 7:** Representative “if-then” rules extracted from the optimized Decision Tree model for malaria risk classification.

ID	Region	Housing & Infra.	Prevention	Socioeconomic	Risk Profile Interpretation
**1**	Busoga	Roof: Grp 1 or 2 Sanitary: Poor	Nets: > 1.5	Rel: ≠ Grandchild	**Ineffective Protection**: High risk in Busoga despite improved roof and nets, likely due to sanitation/usage gap.
**2**	–	Roof: Poor (Grp 3)	Spray: No Nets: None	Asset: Chair Struct: > 1 adult	**Structural Poverty**: Poor housing structure combined with a total lack of vector control.
**3**	≠ Busoga	Roof: Grp 1 or 2 Sanitary: Poor	–	HH Size: > 5.5 Share Bath: No	**Crowding Effect**: Large households coupled with poor sanitation create vulnerability.
**4**	–	Roof: Poor (Grp 3)	Spray: No Nets: None	Asset: No Chair HH Size: > 5.5	**Extreme Deprivation**: Convergence of poor housing, no assets, no protection, and overcrowding.
**5**	–	Roof: Poor (Grp 3)	Spray: No Nets: None	Asset: No Chair Struct: 3 + adults	**Extreme Deprivation (Type B)**: Similar to Rule 4, specific to households with multiple related adults.
**6**	–	Roof: Poor (Grp 3)	Spray: No Nets: None	Asset: No Chair Edu: Primary+	**Environmental Dominance**: Poor housing overrides potential benefits of maternal education.
**7**	Busoga	Roof: Grp 1 or 2	Nets Owned: ≤1.5	–	**Regional Vulnerability**: In Busoga, possessing few nets is a sufficient driver for infection.
**8**	West Nile	Roof: Grp 1 or 2 Sanitary: Imp.	–	–	**Geographic Determinant**: Region is a primary risk factor, overriding better housing.
**9**	–	Roof: Poor (Grp 3)	Spray: No Nets: None	Asset: Chair Struct: 1 adult	**Lone Adult Vulnerability**: Single-adult households with poor structure and no prevention.
**10**	–	Roof: Poor (Grp 3)	Spray: No Nets: None	Asset: No Chair Edu: None	**Cumulative Disadvantage**: The “perfect storm”: poor house, no education, no assets, no protection.

Even in households with improved roof materials (historically a protective factor), the risk of malaria remains high when combined with indicators of extreme poverty-specifically, the absence of a chair and the failure to use mosquito nets. This suggests that structural improvements to housing are insufficient if behavioral barriers (net usage) and deep socioeconomic deprivation persist. For policymakers, this indicates that infrastructure programs in Busoga must be paired with ongoing behavioral change communication and economic support, as “better housing” does not automatically translate into “lower risk” for the poorest families.

The convergence of poor housing structure (thatch/mud roofs), lack of maternal education, and absence of vector control (no spraying or nets) creates a near-certainty of infection. The explicit link to “Mother’s highest educational level” reinforces that malaria control is also an educational issue. In this region, interventions cannot be piecemeal; they require a holistic “package” approach that simultaneously addresses housing, education, and free distribution of nets, as the population lacks the resources to seek protection independently.

Large household size acts as a stressor on limited resources. As seen in Rule 3, even in households without shared bathrooms, the combination of a large family and unprotected water sources creates a high-risk environment. Biologically, higher human density can increase carbon dioxide output and heat, potentially attracting more vectors, while socially, it strains the ratio of available nets per person. This finding validates the need for proportional distribution of nets (e.g., one net per two people) rather than per-household distribution, especially in rural agricultural communities.

Across the generated rules, the hierarchy of risk factors is clear. While ‘Region’ fundamentally determines the baseline transmission intensity, this reflects the pre-existing prevalence in those communities. In high-burden regions like Busoga, the intense baseline transmission pressure significantly lowers the threshold of socioeconomic vulnerability required for infection. Consequently, structural protective factors that might be sufficient elsewhere are overridden, and factors like poor sanitation or insufficient nets become critical sufficient triggers. The specific vulnerability of a child is modulated by the “Asset-Education-Protection” triad:

- Assets: Proxies for wealth (chair, table, roof material).
- Education: Maternal literacy as a gatekeeper for health behaviors.
- Protection: The presence and use of nets and spraying.

Furthermore, maternal education intersects deeply with community health-seeking behaviors. The practical success of infrastructural recommendations can be delayed by behavioral factors, such as the reliance on traditional herbal remedies before seeking formal medical care, highlighting the need for culturally sensitive behavioral change communication.

The interpretability of these rules validates the Decision Tree model not just as a predictor, but as a diagnostic tool for health inequities. The logical flow of this methodology—moving from the dataset validation in Table 4 to the optimized balancing in Table 6—ultimately enabled the extraction of the explicit ‘if-then’ rules detailed in Table 7. This progression highlights that this pipeline was engineered to prioritize a model structure that delivers both high operational performance and deterministic rules. By leveraging an intrinsically interpretable tree, we ensure that public health workers can easily deploy these logical risk profiles offline in resource-constrained field settings.

## Conclusion

This study establishes a framework for utilizing socioeconomic data within machine learning pipelines to inform public health policy for malaria in Uganda. By rigorously comparing algorithms and prioritizing metrics like the F2-score, we demonstrated that standard accuracy is an insufficient metric for epidemiological control. By optimizing Decision Trees and combining them with the RUS technique, we successfully mitigated dataset imbalance, elevating the model’s sensitivity (Recall) to 77%. This ensures a more robust detection of vulnerable children, minimizing false negatives, which are critical in disease outbreaks.

Crucially, our research advances the field by moving beyond prediction accuracy to model interpretability. We derived a set of clear, actionable “if-then” rules that delineate how specific combinations of factors—such as household overcrowding, lack of mosquito nets, and maternal education—hierarchically influence malaria incidence. These interpretable rules provide policymakers with an evidence-based tool to visualize the hierarchical importance of risk factors, enabling the transition from generic prevention strategies to precise, data-driven interventions.

Despite these contributions, this study is subject to certain limitations. First, the cross-sectional nature of the MIS data restricts the ability to infer causality or perform temporal analysis. Furthermore, our approach focuses strictly on socioeconomic determinants and does not account for ongoing biological shifts, such as insecticide resistance or changes in vector species. Consequently, the extracted ‘if-then’ rules represent a snapshot in time and should be periodically retrained with updated survey data to maintain their field accuracy.

Second, although the RUS technique significantly improved the identification of positive cases, the trade-off in overall precision requires that these predictive tools be used as triage mechanisms rather than as stand-alone, definitive diagnostic tools. Because our model relies strictly on socioeconomic proxies rather than clinical biomarkers, these performance metrics would be unacceptable for definitive clinical diagnosis, but they are highly effective for population-level triage. By narrowing the epidemiological search space to profiles capturing nearly 80% of infections, health workers can efficiently direct limited resources—such as tests and bed nets—to high-risk groups rather than relying on random screening.

To achieve the best predictive results and overcome the infrequent nature of national surveys, future research should prioritize the integration of longitudinal (cohort or panel) data. Combining these records with high-resolution spatio-temporal datasets and real-time environmental/climatic variables (such as local rainfall and temperature) would provide a much more dynamic framework for malaria prediction. Additionally, while our explicit rule-based approach provides necessary offline guidelines, future research employing complex ensemble models should integrate SHAP (SHapley Additive exPlanations) analysis to gracefully handle feature interactions. Finally, a critical next step is validating the extracted ‘if-then’ rules in real-world clinical settings and piloting them within community health programs in high-risk regions like Busoga and Karamoja.

## Supporting information

S1 Table Description of socioeconomic and demographic features extracted from the 2018–2019 Uganda Malaria Indicator Survey, including variable categories and dimensional expansion (initial and final column counts) after one-hot encoding pre-processing. (PDF)

S2 Table Detailed hyperparameter configurations for the machine learning algorithms implemented using the Scikit-Learn library for comparative performance analysis. (PDF)

## References

[1] World Health Organization. World Malaria Report 2023. Geneva: World Health Organization. 2023. https://www.who.int/teams/global-malaria-programme/reports/world-malaria-report-2023

[2] National Malaria Control Division, Uganda Bureau of Statistics, ICF. 2018-19 Uganda Malaria Indicator Survey Atlas of Key Indicators. Kampala, Uganda, and Rockville, Maryland, USA: NMCD, UBOS, and ICF. 2019. https://dhsprogram.com/pubs/pdf/ATR21/ATR21.pdf

[3] Mariki M, Mkoba E, Mduma N. Combining clinical symptoms and patient features for malaria diagnosis: machine learning approach. Applied Artificial Intelligence. 2022;36. doi: 10.1080/08839514.2022.2031826

[4] Uddin S, Khan A, Hossain ME, Moni MA. Comparing different supervised machine learning algorithms for disease prediction. BMC Med Inform Decis Mak. 2019;19(1):281. doi: 10.1186/s12911-019-1004-8 31864346 PMC6925840

[5] Jatobá A, de Castro-Nunes P, Palmieri P, Machado Araujo de Oliveira O, Passos Simões P, da Silva Fonseca V, et al. Predictive estimations of health systems resilience using machine learning. BMC Med Inform Decis Mak. 2025;25(1):267. doi: 10.1186/s12911-025-03111-7 40665289 PMC12261563

[6] Coimbra AG, Oliveira CG, Libório MP, Mannan H, Santos LI, Fusco E, et al. Approaches for handling imbalanced data used in machine learning in the healthcare field: A case study on Chagas disease database prediction. PLoS One. 2025;20(5):e0320966. doi: 10.1371/journal.pone.0320966 40359315 PMC12074545

[7] Aglarci AV, Karakurt F. Symptoms affecting the development of diabetes: analysis of risk factors with data mining. BMC Med Inform Decis Mak. 2025;25(1):319. doi: 10.1186/s12911-025-03159-5 40866963 PMC12392476

[8] Barboza MFX, de Carvalho Monteiro KH, Rodrigues IR, Santos GL, Monteiro WM, Figueira ÉAG. Prediction of malaria using deep learning models: A case study on city clusters in the state of Amazonas, Brazil, from 2003 to 2018. Revista da Sociedade Brasileira de Medicina Tropical. 2022;55. doi: 10.1590/0037-8682-0420-2021 PMC9344950 35946631

[9] Semakula M, Niragire F, Faes C. Spatio-Temporal Bayesian Models for Malaria Risk Using Survey and Health Facility Routine Data in Rwanda. Int J Environ Res Public Health. 2023;20(5):4283. doi: 10.3390/ijerph20054283 36901291 PMC10001932

[10] Semakula HM, Liang S, Mukwaya PI, Mugagga F, Nseka D, Wasswa H, et al. Bayesian belief network modelling approach for predicting and ranking risk factors for malaria infections among children under 5 years in refugee settlements in Uganda. Malar J. 2023;22(1):297. doi: 10.1186/s12936-023-04735-8 37794401 PMC10552276

[11] Alexandrov IA, Kuklin VZh, Chervyakov LM, Sheptunov SA. Development of a Technique for Discrete-Logical Decision-Making in Medical Information Systems. HighTech Innov J. 2024;5(4):1008–23. doi: 10.28991/hij-2024-05-04-010

[12] Awe OO, Mwangi PN, Goudoungou SK, Esho RV, Oyejide OS. Explainable AI for enhanced accuracy in malaria diagnosis using ensemble machine learning models. BMC Med Inform Decis Mak. 2025;25(1):162. doi: 10.1186/s12911-025-02874-3 40217281 PMC11987329

[13] Semakula HM, Mugagga F. Predictors of insecticide-treated nets utilization among children under five years in refugee settlements in Uganda: analysis of the 2018-2019 Uganda Malaria Indicator Survey. Malar J. 2025;24(1):20. doi: 10.1186/s12936-025-05262-4 39838479 PMC11752800

[14] Capitoli G, Nobile MS, Ambags EL, L’Imperio V, Provenzano M, Liò P. Assisting clinical diagnosis with interpretable fuzzy probabilistic modelling. BMC Med Inform Decis Mak. 2025;25(Suppl 3):330. doi: 10.1186/s12911-025-03183-5 40954454 PMC12439376

[15] Ferrari D, Arina P, Edgeworth J, Curcin V, Guidetti V, Mandreoli F, et al. Using interpretable machine learning to predict bloodstream infection and antimicrobial resistance in patients admitted to ICU: Early alert predictors based on EHR data to guide antimicrobial stewardship. PLOS Digit Health. 2024;3(10):e0000641. doi: 10.1371/journal.pdig.0000641 39413052 PMC11482717

[16] Pedregosa F, Varoquaux G, Gramfort A, Michel V, Thirion B, Grisel O. Scikit-learn: Machine learning in Python. Journal of Machine Learning Research. 2011;12:2825–30.

[17] Toygar İ, Özgür S, Bağçivan G, Karaçam E, Benzer H, Özdemir FA, et al. A machine learning approach to predict self-efficacy in breast cancer survivors. BMC Med Inform Decis Mak. 2025;25(1):313. doi: 10.1186/s12911-025-03155-9 40830946 PMC12366306

[18] Sharma A, Verbeke WJMI. Understanding importance of clinical biomarkers for diagnosis of anxiety disorders using machine learning models. PLoS One. 2021;16(5):e0251365. doi: 10.1371/journal.pone.0251365 33970950 PMC8109802

[19] Ning Y, Li S, Ng YY, Chia MYC, Gan HN, Tiah L, et al. Variable importance analysis with interpretable machine learning for fair risk prediction. PLOS Digit Health. 2024;3(7):e0000542. doi: 10.1371/journal.pdig.0000542 38995879 PMC11244764

[20] D’Abramo A, Rinaldi F, Vita S, Mazzieri R, Corpolongo A, Palazzolo C, et al. A machine learning approach for early identification of patients with severe imported malaria. Malar J. 2024;23(1):46. doi: 10.1186/s12936-024-04869-3 38351021 PMC10865572

[21] Zaloumis SG, Rajasekhar M, Simpson JA. How to use learning curves to evaluate the sample size for malaria prediction models developed using machine learning algorithms. Malar J. 2025;24(1):242. doi: 10.1186/s12936-025-05479-3 40708012 PMC12291394

[22] Khan O, Ajadi JO, Hossain MP. Predicting malaria outbreak in The Gambia using machine learning techniques. PLoS One. 2024;19(5):e0299386. doi: 10.1371/journal.pone.0299386 38753678 PMC11098333

[23] Chicco D, Jurman G. The Matthews correlation coefficient (MCC) should replace the ROC AUC as the standard metric for assessing binary classification. BioData Mining. 2023;16:4–4. doi: 10.1186/s13040-023-00322-4 36800973 PMC9938573

[24] Lemaître G, Nogueira F, Aridas CK. Imbalanced-learn: A python toolbox to tackle the curse of imbalanced datasets in machine learning. Journal of Machine Learning Research. 2017;18(17):1–5.

[25] Chawla NV, Bowyer KW, Hall LO, Kegelmeyer WP. SMOTE: Synthetic Minority Over-sampling Technique. jair. 2002;16:321–57. doi: 10.1613/jair.953

