# Supplementary material for: Socioeconomic determinants of malaria in Ugandan children: An interpretable machine learning approach for public health policy
Source: PLOS Digit Health. 2026 Sep 3;5(9):e0001152. doi: 10.1371/journal.pdig.0001152 (PMC13541157; doi:10.1371/journal.pdig.0001152)
Supplement: Supplementary file 1 [file pdig.0001152.s001.pdf]

Supplementary Table 1. Description of socioeconomic and demographic features extracted from the 2018-2019 Uganda Malaria Indicator Survey, including variable categories and dimensional expansion (initial and final column counts) after one-hot encoding pre-processing.

**Features**	**Values**	**Initial**	**Final**
Total number of household members	2 to 36	1	1
Number of resident children under 5 years old	1 to 9	1	1
Region of residence	West Nile, Acholi, Tooro, Bunyoro, Lango, South Buganda, Teso, Karamoja, Busoga, Bukedi, North Buganda, Ankole, Bugisu, Kigezi, Kampala	1	15
Type of region	Urban, rural, or refugee area	1	3
Main source of drinking water	**Group 1:** 'Piped into dwelling', 'Piped to yard/plot', 'Public tap/standpipe', 'Tube well or borehole', 'Protected well', 'Protected spring', 'Rainwater', 'Bottled water', 'Sachet water' **Group 2:** 'Piped to neighbor', 'Tanker truck', 'Bicycle with jerrycans' **Group 3:** 'Unprotected well', 'Unprotected spring', 'River/dam/lake/ponds/stream/canal/irrigation channel'	1	3
Main type of cooking fuel	**Group 1:** 'Electricity', 'Biogas', 'LPG/Cylinder gas' **Group 2:** 'Charcoal', 'Kerosene' **Group 3:** 'Wood', 'Agricultural crop', 'Straw/shrubs/grass', 'No food cooked in house'	1	3
Main floor material	**Group 1:** 'Cement', 'Concrete', 'Carpet', 'Ceramic tiles', 'Parquet or polished wood' **Group 2:** 'Stones', 'Bricks', 'Wood planks' **Group 3:** 'Dung', 'Earth/sand', 'Palm/bamboo'	1	3
Main wall material	**Group 1:** 'Cement', 'Cement blocks', 'Burnt bricks with cement', 'Stone with lime/cement' **Group 2:** 'Unburnt bricks with plaster', 'Unburnt bricks with cement', 'Burnt bricks with mud', 'Stone with mud', 'Unburnt bricks with mud', 'Poles with mud' **Group 3:** 'Wood planks/shingles', 'Reused wood', 'Plywood', 'Thatched/straw', 'Cardboard', 'Dirt', 'No walls', 'Other'	1	3

Continued on next page

Features	Values	Initial	Final
Main roof material	**Group 1:** 'Concrete', 'Tiles', 'Asbestos', 'Roofing shingles', 'Iron sheets' **Group 2:** 'Wood planks', 'Wood' **Group 3:** 'Thatch/palm leaf', 'Rustic mat', 'Mud', 'Cardboard', 'Tarpaulin', 'No roof'	1	3
Type of sanitary facility	**Group 1:** 'Flush to piped sewer system', 'Flush to septic tank', 'Flush to pit latrine', 'Ventilated Improved Pit latrine (VIP)', 'Pit latrine with slab', 'Composting toilet' **Group 2:** 'Flush, don't know where', 'Flush to somewhere else' **Group 3:** 'Pit latrine without slab/open pit', 'Hanging toilet/latrine', 'No facility/bush/field', 'Other'	1	3
If the household shares a bathroom with other households	Yes or No	1	1
If the household has: electricity, radio, television, refrigerator, bicycle, motorcycle, car, landline telephone, cell phone, clock, cart drawn by animals, boat with engine, boat without engine, computer, land suitable for agriculture, cassette/CD/DVD player, table, chair, sofa set, bed, closet, clock, animals, if at least one resident has a bank account	Yes or No	24	24
Household structure relationship	Two adults, opposite sex, Two adults, same sex, Three+ related adults, One adult, Unrelated adults	1	5
Number of rooms used for sleeping	1 to 10	1	1
If the home has at least one mosquito net	Yes or No	1	1
Number of mosquito nets the household owns	0 to 7	1	1

Continued on next page

Features	Values	Initial	Final
Number of children sleeping under a mosquito net last night	0 to 6	1	1
If children sleep under a mosquito net	All children, No, No net in household, Some children	1	1
Dwelling sprayed against mosquitoes in the last 12 months, Spraying done: by government, private company, NGO, Other	Yes or No	5	5
Relationship to the head of the household	Son/daughter, Other relative, Grandchild, Adopted/foster/stepchild, Brother/sister, Son/daughter-in-law, Not related	1	7
If the member is a permanent resident, If the member is a usual resident	Yes or No	2	2
Mother's highest educational level	Secondary, Primary, Higher, No education	1	1
Type of mosquito net the person slept in last night	Only treated nets, Did not sleep under a net, Only untreated nets	1	3
Sleep under a LLIN mosquito net	Yes or No	1	1
Sleep under a mosquito net that is always treated	Yes or No	1	1
Malaria blood test result	Negative or Positive	1	1
**Total**		**54**	**94**
