# Supplementary material for: Socioeconomic determinants of malaria in Ugandan children: An interpretable machine learning approach for public health policy
Source: PLOS Digit Health. 2026 Sep 3;5(9):e0001152. doi: 10.1371/journal.pdig.0001152 (PMC13541157; doi:10.1371/journal.pdig.0001152)
Supplement: Supplementary file 2 [file pdig.0001152.s002.pdf]

Supplementary Table 2. Detailed hyperparameter configurations for the machine learning algorithms implemented using the Scikit-Learn library for comparative performance analysis.

Class	Parameters
LogisticRegression	penalty='l2', dual=False, tol=0.0001, C=1.0, fit_intercept=True, intercept_scaling=1, class_weight=None, random_state=None, solver='lbfgs', max_iter=100, multi_class='deprecated', verbose=0, warm_start=False, n_jobs=None, l1_ratio=None
SGDClassifier	loss='log-loss', penalty='l2', alpha=0.0001, l1_ratio=0.15, fit_intercept=True, max_iter=1000, tol=0.001, shuffle=True, verbose=0, epsilon=0.1, n_jobs=None, random_state=0, learning_rate='optimal', eta0=0.0, power_t=0.5, early_stopping=False, validation_fraction=0.1, n_iter_no_change=5, class_weight=None, warm_start=False, average=False
SVC	C=1.0, kernel='sigmoid', degree=3, gamma='scale', coef0=0.0, shrinking=True, probability=True, tol=0.001, cache_size=200, class_weight=None, verbose=False, max_iter=-1, decision_function_shape='ovr', break_ties=False, random_state=0
DecisionTreeClassifier	criterion='gini', splitter='best', max_depth=None, min_samples_split=2, min_samples_leaf=1, min_weight_fraction_leaf=0.0, max_features=None, random_state=0, max_leaf_nodes=None, min_impurity_decrease=0.0, class_weight=None, ccp_alpha=0.0, monotonic_cst=None
RandomForestClassifier	n_estimators=100, criterion='gini', max_depth=None, min_samples_split=2, min_samples_leaf=1, min_weight_fraction_leaf=0.0, max_features='sqrt', max_leaf_nodes=None, min_impurity_decrease=0.0, bootstrap=True, oob_score=False, n_jobs=None, random_state=0, verbose=0, warm_start=False, class_weight=None, ccp_alpha=0.0, max_samples=None, monotonic_cst=None
GradientBoostingClassifier	loss='log_loss', learning_rate=0.1, n_estimators=100, subsample=1.0, criterion='friedman_mse', min_samples_split=2, min_samples_leaf=1, min_weight_fraction_leaf=0.0, max_depth=3, min_impurity_decrease=0.0, init=None, random_state=0, max_features=None, verbose=0, max_leaf_nodes=None, warm_start=False, validation_fraction=0.1, n_iter_no_change=None, tol=0.0001, ccp_alpha=0.0
MLPClassifier	hidden_layer_sizes=(100,), activation='relu', *, solver='adam', alpha=0.0001, batch_size='auto', learning_rate='constant', learning_rate_init=0.001, power_t=0.5, max_iter=1000, shuffle=True, random_state=None, tol=0.0001, verbose=False, warm_start=False, momentum=0.9, nesterovs_momentum=True, early_stopping=False, validation_fraction=0.1, beta_1=0.9, beta_2=0.999, epsilon=1e-08, n_iter_no_change=10, max_fun=15000
KNeighborsClassifier	n_neighbors=3, weights='uniform', algorithm='auto', leaf_size=30, p=2, metric='minkowski', metric_params=None, n_jobs=None
GaussianNB	priors=None, var_smoothing=1e-09
