# Supplementary material for: Socioeconomic determinants of malaria in Ugandan children: An interpretable machine learning approach for public health policy
Source: PLOS Digit Health. 2026 Sep 3;5(9):e0001152. doi: 10.1371/journal.pdig.0001152 (PMC13541157; doi:10.1371/journal.pdig.0001152)
Supplement: Supplementary file 3 [file pdig.0001152.s005.pdf]

Response to Reviewers

Manuscript Title: Socioeconomic Determinants of Malaria in Ugandan Children: An Interpretable Machine Learning Approach for Public Health Policy
Journal: PLOS Digital Health

Dear Editor and Reviewers,

We would like to express our sincere gratitude for your time, dedication, and highly constructive feedback on our manuscript. We truly appreciate the thorough evaluation and the valuable insights provided, which have significantly strengthened the methodological clarity, structural flow, and practical implications of our work.

In this document, we present a detailed, point-by-point response to each of the comments and questions raised. For your convenience, the reviewers' original comments are listed below, followed directly by our responses and explanations of the specific changes made to the text.

Additionally, to facilitate the tracking of these modifications in the revised manuscript, **all newly added text is highlighted in blue**, while any **deleted text is presented with a ~~strikethrough~~ format**.

Thank you once again for your guidance and for helping us improve the quality of this research.

Sincerely,

The Authors

Journal Requirement: Inclusion of an Author Summary
Response: In accordance with the editorial guidelines of PLOS Digital Health, we have added an "Author Summary" to the revised manuscript. We crafted a 182-word, non-technical summary written in the first-person voice, avoiding complex terminology to ensure our findings and their public health implications are accessible to a broader audience, including non-scientists and policymakers. The new "Author Summary" has been inserted immediately following the Abstract and just before the Introduction section in the revised manuscript. The added text is highlighted in blue for easy identification.

Reviewer #1:

Point 1

Dear Authors,
I truly appreciate your methodology, especially the choice to prioritize the F2-score. Focusing on finding as many infected children as possible is vital for saving lives. However, I would like to seek clarity on a few points:
• Regional Trends: Your results show the Busoga region standing out as a high-risk area. Since other records show Busoga already has a very high baseline malaria prevalence (21.1%), could you clarify if this existing high burden significantly influenced how the "if-then" rules were formed for that area?

Our answer:

Thank you for your insightful question and for recognizing the value of prioritizing the F2-score in our methodology. You are entirely correct in your observation. The existing high burden of malaria in the Busoga region significantly influenced how the decision tree model formulated the “if-then” rules for that specific area. In our analysis, we discovered that the feature “Region” fundamentally determines the intensity of basal transmission. We have clarified this interaction in the revised manuscript by adding a paragraph in the subsection “Generation of rules from the Decision Tree”.

Point 2

• *Data Balancing: You used Random Under-Sampling (RUS) to balance your data. While this helped find more cases, it also dropped the overall accuracy to 61%. Do you worry that the resulting false positives might overwhelm healthcare workers who are already facing limited resources?*

Our answer:
Thank you for raising this highly relevant point regarding healthcare resource constraints. We share your concern that excessive false positives could burden the system; however, we approached this challenge by evaluating the highly asymmetric cost of misclassification in public health. In the context of malaria in children under five, a False Negative (failing to identify an infected child) carries a severe risk of morbidity, mortality, and perpetuates the transmission reservoir. On the other hand, a False Positive (classifying a healthy child as high-risk) primarily incurs an economic cost—specifically, the cost of a confirmatory Rapid Diagnostic Test (RDT). Because malaria is a life-threatening condition, minimizing False Negatives was treated as our ethical priority.
To address the potential overwhelm of healthcare workers, it is crucial to position our Decision Tree model not as a standalone, definitive diagnostic tool, but as a first-line triage mechanism. Rather than increasing the burden, the model actually optimizes limited resources by filtering the population. By identifying specific socioeconomic profiles, health workers can direct limited RDTs and bed net distribution campaigns exclusively to these high-risk groups, which is far more efficient than random screening. The model effectively narrows the search space, ensuring that the vast majority of infected children are flagged for intervention, making the cost of testing some non-infected children an acceptable and necessary trade-off. We have ensured this distinction is more clearly articulated in the manuscript by adding a discussion in the subsection “Decision Tree analysis” and in the “Conclusion” of the work.

Point 3

• *Rule Practicality: Finally, how do you see these socioeconomic rules holding up against biological shifts like insecticide resistance or changes in mosquito species? Additionally, since studies show about 21.2% of people still use local herbal remedies before seeking medical care, how might these behavioural factors affect the success of your recommendations in the field?*

Our answer:

Thank you for bringing up these excellent and highly practical points regarding the dynamic nature of malaria transmission and community health-seeking behaviors. Regarding your first point on **biological shifts**, you are entirely correct. Our current model provides an epidemiological snapshot based on the cross-sectional data from the 2018-2019 MIS. Because our approach focuses strictly on socioeconomic determinants, it does not account for ongoing biological and behavioral features. We anticipate that as these biological and behavioral shifts occur, the specific thresholds within our "if-then" rules (such as Rule 7) will inevitably shift. Therefore, we view our Decision Tree framework not as a static set of rules, but rather as a dynamic methodological tool that should be periodically retrained with updated survey data to reflect the evolving reality on the ground. We have made this perception clearer in the revised manuscript by adding a discussion in the section “Generation of rules from the Decision Tree” and expanding the limitations discussed in the Conclusion.

Reviewer #2:

Point 1

Minor Points
Introduction
1. The paragraph starting in line 59 where you talk about the data being used in the study feels misplaced. Wouldn’t rather be placed in the “materials and methods” section?
Materials and Methods

Our answer:
Thank you for pointing this out; we completely agree with your observation. We have removed this paragraph from the Introduction to improve the flow of the text. We also ensured that all specific details previously mentioned there were fully integrated into the 'Materials and Methods' section

Point 2

2. 112 - The title of table 1 is not self-explanatory. Make it more descriptive so that a person can understand without referring elsewhere what the table is all about.

Our answer:
Thank you for this highly constructive feedback. We completely agree that the original title ('Features overview') was too brief and lacked the necessary context for a standalone interpretation. We have updated the title of Table 1 to make it fully self-explanatory “Description of socioeconomic and demographic features extracted from the 2018-2019 Uganda Malaria Indicator Survey, including variable categories and dimensional expansion (initial and final column counts) after one-hot encoding pre-processing.”

Point 3

3. 175 - Table 2 as well should have a self-descriptive title.
Results

Our answer:
Thank you for pointing this out. We completely agree that Table 2, much like Table 1, required a more comprehensive and self-explanatory title. We have updated the title of Table 2 to: “Detailed hyperparameter configurations for the machine learning algorithms implemented using the Scikit-Learn library for comparative performance analysis.” We appreciate your attention to detail, which helps make our manuscript much clearer for independent reading.

Point 4

4. Line 325 – why did you choose 10 rules out of 58? What method did you use to pick only 10?
Conclusion

Our answer:
Thank you for this highly pertinent question regarding our rule selection methodology. We agree that the criteria for selecting the 10 rules out of the 58 generated could be more explicitly stated in the text. It was our failure to clearly explain the choice of rules in the manuscript. To guarantee the interpretability and practical application of these findings, we applied specific structural filtering criteria. Out of the 58 rules generated, we exclusively selected those that predicted the target class and that were at most 6 levels deep. This depth restriction ensures that the 'if-then' conditions remain cognitively accessible and actionable for public health officials. We have updated the text to clarify this methodological choice adding a explanation in the section “Generation of rules from the Decision Tree”.

Point 5

5. 376 – Great that you acknowledge limitations in the MIS data used (the cross-sectional nature of it). It would be important to give recommendations on the type of data that would give the best results.
Confidential Comments to the editor
Needs minor revisions.

Our answer:
Thank you for your encouraging feedback and for highlighting this important point. We completely agree that simply acknowledging the limitations of the cross-sectional MIS data is not enough, and that explicitly recommending the ideal types of data for future studies adds significant value and direction to the manuscript. To address your comment, we have expanded the limitations paragraph in the Conclusion section.

Reviewer #3:

Point 1

Review Notes
Comments on Methods:
Feature Importance using Random Forest
Random forests calculate feature importance in two main ways:
Mean Decrease in Impurity (MDI) - Also called "Gini importance," this measures how much each feature decreases the weighted impurity (like Gini impurity or entropy) across all trees in the forest. Features that split nodes with higher impurity reduction are considered more important.
Mean Decrease in Accuracy (MDA) - Also called "permutation importance," this measures how much the model's accuracy drops when you randomly shuffle the values of a feature. If shuffling a feature causes a big drop in accuracy, that feature is important.
One thing to keep in mind is that random forest feature importance can be biased toward high-cardinality features (features with many unique values) and correlated features can have their importance split between them. For more robust importance estimates, permutation importance is often used.
Usually Random Forest's built-in importance is used when one needs a quick sanity check during exploratory analysis and don't need rigorous interpretation.
My recommendation is to use SHAP ((SHapley Additive exPlanations) analysis
SHAP values offer several advantages:
They're theoretically grounded (based on cooperative game theory) and provide consistent, locally accurate explanations
They explain both global importance and individual predictions
They handle feature interactions and correlations more gracefully
They work with any model, not just tree-based ones
LIME (Local Interpretable Model-agnostic Explanations) is another excellent interpretability tool but for this project SHAP will be the best.

Our answer:
Thank you for this excellent and highly technical recommendation. We completely agree that SHAP values represent the state-of-the-art for model interpretability, offering robust, theoretically grounded explanations that beautifully handle feature interactions.

However, we would like to clarify the specific role of the Random Forest in our methodology. We did not use the Random Forest’s built-in importance for the final rigorous interpretation of the epidemiology. Instead, it was employed strictly as an initial dimensionality reduction step (feature selection) to filter the features from 93 down to 37. The core interpretability of our study—which public health officials need for actionable insights—was derived from the explicit 'if-then' rules generated by the optimized single Decision Tree.

Furthermore, regarding the well-known bias of Mean Decrease in Impurity (MDI) toward high-cardinality features, our pre-processing step largely mitigated this issue. Before applying the Random Forest, all categorical variables were transformed using one-hot encoding. Because the algorithm was fed almost exclusively binary features, the cardinality bias was significantly neutralized.

Nevertheless, your point is extremely valid for advancing this research. We have updated our methodology section, specifically “Feature selection using Random Forest”, to clarify how the one-hot encoding mitigated the MDI bias during feature selection. We have also added a recommendation in the Conclusion that future studies employing complex ensemble models should adopt SHAP values to provide both local and global explanations, complementing our rule-based approach.

Point 2

Machine Learning Methods:
There is no need to compare machine learning models if their performance is not significantly different. My recommendation is to use decision tress, random forest, neural networks and other deep learning methods. I understand that the objective here is to come up with an interpretable model. But using SHAP we can determine best model irrespective of their characteristics (opaque or transparent). Table 6 performance figure do not show much improvement (model by model). If the features were clinical and not socio economic indicators then this model will not work due to unacceptable recall values and F1 score.

Our answer:
Thank you for your critical and thought-provoking comments, which allow us to further clarify the methodological rationale of our study. Regarding your first point on the models evaluated, we would like to highlight that our comparative analysis (Table 6) already includes the exact models you recommended: Decision Trees, Random Forest, and a Neural Network (MLPClassifier). The purpose of Table 6 was precisely to demonstrate the 'accuracy paradox' in its baseline state—showing that complex models like Gradient Boosting or Neural Networks might achieve high accuracy but yield unacceptably low Recall (e.g., 6% or 20%) before data balancing is applied. The lack of significant improvement model-by-model at this baseline stage was the exact justification for our subsequent step: applying balancing techniques (like RUS) to our chosen model, which successfully elevated the Recall to a much more robust 77% (Table 8).

Regarding your recommendation to use SHAP to determine the best model regardless of its opaque nature, we completely agree that SHAP is the gold standard for explainable AI. However, there is a fundamental difference between post-hoc explainability (SHAP) and inherent interpretability (Decision Trees). In the context studied, our goal was to generate explicit, standalone 'if-then' rules. A single decision tree provides a physical, logical flowchart (as seen in Table 9) that a policymaker or field worker can understand and apply 'offline' without needing a computer, a Python environment, or an inference engine running a SHAP explainer. We have added a note in the manuscript clarifying why inherent interpretability was prioritized over post-hoc explainers for this specific deployment context.

Finally, we do not propose this model as a clinical diagnostic tool to replace microscopy or rapid tests. As stated in our manuscript, because we are using socioeconomic determinants, this model is strictly designed as a first-line public health triage mechanism. Its purpose is to map vulnerability and direct resource allocation (like bed nets and targeted testing), making a 77% recall highly valuable for narrowing the search space in population-level screening.

We have reinforced this crucial distinction between clinical diagnosis and socioeconomic triage in the conclusion, we added a discussion in the subsection “Comparison of machine learning algorithms” and “Decision Tree analysis”.

Point 3

Restructuring the article:
This is important work. I recommend restructuring the article in the following format
Background
Data
Methods (directly apply decision trees, random forest, and neural networks)
Methods (SHAP analysis)
Results and discussions (feature importance using SHAP and just report performance for best model. Need not include all the model results)
Appendix
The authors need to mention all model hyperparameters in appendix section. Table and and Table 2 can be added to appendix section than in the main article.

Our answer:

Thank you for your thoughtful recommendations regarding the structure of our manuscript. We appreciate your guidance on improving the flow and readability of the article. We adopted your suggestion regarding the presentation of data and hyperparameters. The extensive Table 1 and Table 2 have been moved to an Appendix section at the end of the manuscript. We have also restructured our titles to better separate the 'Data' and 'Methods' subsections, aligning them with the suggested flow.

Regarding your recommendation to structure the Methods and Results around a SHAP analysis and report only the performance of the best model, we must respectfully maintain our current approach due to the specific public health objectives of this study and the considerations of the other reviewers. We believe that keeping the comprehensive model comparison and the explicit rule generation, combined with your excellent structural suggestion to move the heavy tables to the Appendix, provides the most robust and accessible version of this research. Thank you for helping us optimize the presentation of our work.
