# Supplementary material for: Socioeconomic determinants of malaria in Ugandan children: An interpretable machine learning approach for public health policy
Source: PLOS Digit Health. 2026 Sep 3;5(9):e0001152. doi: 10.1371/journal.pdig.0001152 (PMC13541157; doi:10.1371/journal.pdig.0001152)
Supplement: Supplementary file 4 [file pdig.0001152.s006.pdf]

Response to Reviewers

Manuscript Title: Socioeconomic Determinants of Malaria in Ugandan Children: An Interpretable Machine Learning Approach for Public Health Policy
Journal: PLOS Digital Health

Dear Editor and Reviewers,
We would like to express our sincere gratitude to the Editorial Board and the Reviewers for their time, dedication, and highly constructive feedback on our manuscript. We truly appreciate the thorough evaluation, as the insightful suggestions provided have significantly strengthened the methodological clarity, structural flow, and practical relevance of our study.
Below, we present a detailed, point-by-point response addressing each comment and question raised. For clarity, the original comments are listed in sequence, followed immediately by our explicit responses and a description of the specific revisions incorporated into the updated text.

Additionally, to facilitate the tracking of these modifications in the revised manuscript, **all newly added text is highlighted in blue**, while any **deleted text is presented with a ~~strikethrough~~ format**.

Thank you once again for your guidance and for helping us improve the quality of this research.

Sincerely,

The Authors

Additional Editor Comments
"*The paper has been well improved. The title is An Interpretable Machine Learning Approach, however the main interpretable algorithm proposed was decision tree (DT), which is a classic ML algorithm that was proposed many decades ago. More important, DT was compared to a group of other ML algorithms, and its performance is not the best. It would be better to make the best model (in table 4) interpretable/explainable, using SHAP or other tools to enhance the interpretability/expandability.* "

Our answer
Thank you for the constructive feedback on our manuscript, "Socioeconomic Determinants of Malaria in Ugandan Children: An Interpretable Machine Learning Approach for Public Health Policy". Before submitting our formal revision, we would like to respectfully clarify a structural point in our methodology to ensure our upcoming response aligns perfectly with your expectations.
In the section "Comparison of machine learning algorithms," the comparative analysis in Table 4 aimed strictly to validate whether our socioeconomic dataset possessed sufficient predictive capacity for malaria surveillance before advancing the pipeline. Once the data's efficiency was confirmed, our core objective was to develop a highly interpretable alternative rather than relying on complex black-box models. As detailed in the subsequent sections, we addressed the baseline Decision Tree's limitations by optimizing its hyperparameters and applying Random Under-Sampling (RUS) to tackle the severe class imbalance. This tailored

approach allowed the Decision Tree to achieve a 77% Recall and a 49% F2-score (as shown in Table 6), drastically outperforming the complex baseline models from Table 4, such as Gradient Boosting, which collapsed to a 6% Recall due to the accuracy paradox.
This optimized model ultimately enabled the extraction of the explicit "if-then" rules detailed in Table 7. We intentionally prioritized the inherent interpretability of a single tree over post-hoc explainability tools like SHAP so that public health workers can easily deploy these logical risk profiles offline in resource-constrained field settings, without requiring a computational inference engine.
We have integrated the requested clarification directly into the text to explicitly detail our methodological choices for the reader. A new paragraph has been added at the end of the baseline algorithm comparison section “Comparison of machine learning algorithms” to explain that Table 4 functioned strictly as a validation step for the dataset's predictive capacity, and to highlight why the pipeline advanced toward an optimized Decision Tree rather than complex black-box models. Additionally, we reinforced this logical flow at the end of the rule generation section “Generation of rules from the Decision Tree” to demonstrate how the performance gains led to actionable field deployment.
