# Supplementary material for: Socioeconomic determinants of malaria in Ugandan children: An interpretable machine learning approach for public health policy
Source: PLOS Digit Health. 2026 Sep 3;5(9):e0001152. doi: 10.1371/journal.pdig.0001152 (PMC13541157; doi:10.1371/journal.pdig.0001152)
Supplement: Supplementary file 5 [file pdig.0001152.s004.docx]

Dear Editor,

I have carefully gone through the revised version of the manuscript and noted that the authors have adequately addressed each and every comment and concern I raised during the previous review process. The revisions have improved the clarity and quality of the manuscript.

I am satisfied with the responses provided and would recommend the manuscript for publication.

Kind regards,

Daniel Maina Nderitu
