# Supplementary material for: Socioeconomic determinants of malaria in Ugandan children: An interpretable machine learning approach for public health policy
Source: PLOS Digit Health. 2026 Sep 3;5(9):e0001152. doi: 10.1371/journal.pdig.0001152 (PMC13541157; doi:10.1371/journal.pdig.0001152)
Supplement: Peer review history

# Peer review: Decision Letter 0

**DOI:** 10.1371/journal.pdig.0001152.r001

**Authors:** Sandra Barteit, Kezhi Li

9 Mar 2026

Response to Reviewers Revised Manuscript with Track Changes Manuscript **Journal Requirements:**

1.  We ask that a manuscript source file is provided at Revision. Please upload your manuscript file as a .doc, .docx, .rtf or .tex.

2. Please provide an Author Summary. This should appear in your manuscript between the Abstract (if applicable) and the Introduction, and should be 150–200 words long. The aim should be to make your findings accessible to a wide audience that includes both scientists and non-scientists. Sample summaries can be found on our website under Submission Guidelines: [LINK]

https://journals.plos.org/digitalhealth/s/submission-guidelines#loc-parts-of-a-submission

**Additional Editor Comments (if provided):** **Reviewers' Comments:**

**Comments to the Author**

1. Does this manuscript meet PLOS Digital Health’s publication criteria?

Reviewer #1: Yes

Reviewer #2: Yes

Reviewer #3: Partly

**********

2. Has the statistical analysis been performed appropriately and rigorously?

Reviewer #1: Yes

Reviewer #2: Yes

Reviewer #3: No

**********

3. Have the authors made all data underlying the findings in their manuscript fully available (please refer to the Data Availability Statement at the start of the manuscript PDF file)?

The PLOS Data policy

Reviewer #1: Yes

Reviewer #2: Yes

Reviewer #3: Yes

**********

4. Is the manuscript presented in an intelligible fashion and written in standard English?

Reviewer #1: Yes

Reviewer #2: Yes

Reviewer #3: Yes

**********

Reviewer #1: Dear Authors,

I truly appreciate your methodology, especially the choice to prioritize the F2-score. Focusing on finding as many infected children as possible is vital for saving lives. However, I would like to seek clarity on a few points:

• Regional Trends: Your results show the Busoga region standing out as a high-risk area. Since other records show Busoga already has a very high baseline malaria prevalence (21.1%), could you clarify if this existing high burden significantly influenced how the "if-then" rules were formed for that area?

• Data Balancing: You used Random Under-Sampling (RUS) to balance your data. While this helped find more cases, it also dropped the overall accuracy to 61%. Do you worry that the resulting false positives might overwhelm healthcare workers who are already facing limited resources?

• Rule Practicality: Finally, how do you see these socioeconomic rules holding up against biological shifts like insecticide resistance or changes in mosquito species? Additionally, since studies show about 21.2% of people still use local herbal remedies before seeking medical care, how might these behavioural factors affect the success of your recommendations in the field?

Reviewer #2: Minor Points

Introduction

1. The paragraph starting in line 59 where you talk about the data being used in the study feels misplaced. Wouldn’t rather be placed in the “materials and methods” section?

Materials and Methods

2. 112 - The title of table 1 is not self-explanatory. Make it more descriptive so that a person can understand without referring elsewhere what the table is all about.

3. 175 - Table 2 as well should have a self-descriptive title.

Results

4. Line 325 – why did you choose 10 rules out of 58? What method did you use to pick only 10?

Conclusion

5. 376 – Great that you acknowledge limitations in the MIS data used (the cross-sectional nature of it). It would be important to give recommendations on the type of data that would give the best results.

Confidential Comments to the editor

Needs minor revisions.

Reviewer #3: Review Notes

Comments on Methods:

Feature Importance using Random Forest

Random forests calculate feature importance in two main ways:

Mean Decrease in Impurity (MDI) - Also called "Gini importance," this measures how much each feature decreases the weighted impurity (like Gini impurity or entropy) across all trees in the forest. Features that split nodes with higher impurity reduction are considered more important.

Mean Decrease in Accuracy (MDA) - Also called "permutation importance," this measures how much the model's accuracy drops when you randomly shuffle the values of a feature. If shuffling a feature causes a big drop in accuracy, that feature is important.

One thing to keep in mind is that random forest feature importance can be biased toward high-cardinality features (features with many unique values) and correlated features can have their importance split between them. For more robust importance estimates, permutation importance is often used.

Usually Random Forest's built-in importance is used when one needs a quick sanity check during exploratory analysis and don't need rigorous interpretation.

My recommendation is to use SHAP ((SHapley Additive exPlanations) analysis

SHAP values offer several advantages:

They're theoretically grounded (based on cooperative game theory) and provide consistent, locally accurate explanations

They explain both global importance and individual predictions

They handle feature interactions and correlations more gracefully

They work with any model, not just tree-based ones

LIME (Local Interpretable Model-agnostic Explanations) is another excellent interpretability tool but for this project SHAP will be the best.

Machine Learning Methods:

There is no need to compare machine learning models if their performance is not significantly different. My recommendation is to use decision tress, random forest, neural networks and other deep learning methods. I understand that the objective here is to come up with an interpretable model. But using SHAP we can determine best model irrespective of their characteristics (opaque or transparent). Table 6 performance figure do not show much improvement (model by model). If the features were clinical and not socio economic indicators then this model will not work due to unacceptable recall values and F1 score.

Restructuring the article:

This is important work. I recommend restructuring the article in the following format

Background

Data

Methods (directly apply decision trees, random forest, and neural networks)

Methods (SHAP analysis)

Results and discussions (feature importance using SHAP and just report performance for best model. Need not include all the model results)

Appendix

The authors need to mention all model hyperparameters in appendix section. Table and and Table 2 can be added to appendix section than in the main article.

**********

what does this mean?). If published, this will include your full peer review and any attached files.

**Do you want your identity to be public for this peer review?**  If you choose “no”, your identity will remain anonymous but your review may still be made public.

For information about this choice, including consent withdrawal, please see our Privacy Policy

Reviewer #1: No

Reviewer #2: **Yes:** Daniel Maina Nderitu

Reviewer #3: No

**********

**Figure resubmission:**

**Reproducibility:**

To enhance the reproducibility of your results, we recommend that authors of applicable studies deposit laboratory protocols in protocols.io, where a protocol can be assigned its own identifier (DOI) such that it can be cited independently in the future. Additionally, PLOS ONE offers an option to publish peer-reviewed clinical study protocols. Read more information on sharing protocols at https://plos.org/protocols?utm_medium=editorial-email&utm_source=authorletters&utm_campaign=protocols

# Author comment: Author response to Decision Letter 1

**DOI:** 10.1371/journal.pdig.0001152.r002

2 Apr 2026

Attachment Submitted filename: Response to Reviewers.pdf

# Peer review: Decision Letter 1

**DOI:** 10.1371/journal.pdig.0001152.r003

**Authors:** Kezhi Li

30 Jun 2026

Response to ReviewersRevised Manuscript with Track ChangesManuscript**Journal Requirements:** **Additional Editor Comments (if provided):** **Reviewers' Comments:**

**Comments to the Author**

Reviewer #1: All comments have been addressed

Reviewer #2: All comments have been addressed

**********

publication criteria?

Reviewer #1: Yes

Reviewer #2: Yes

**********

3. Has the statistical analysis been performed appropriately and rigorously?

Reviewer #1: Yes

Reviewer #2: Yes

**********

4. Have the authors made all data underlying the findings in their manuscript fully available (please refer to the Data Availability Statement at the start of the manuscript PDF file)?

The PLOS Data policy

Reviewer #1: Yes

Reviewer #2: Yes

**********

5. Is the manuscript presented in an intelligible fashion and written in standard English?

Reviewer #1: Yes

Reviewer #2: Yes

**********

Reviewer #1: (No Response)

Reviewer #2: I have carefully reviewed the revised version of the manuscript and noted that the authors have adequately addressed all the comments and concerns raised during the previous review process. The revisions have significantly improved the clarity and overall quality of the manuscript.

In addition, the dataset used in the study is publicly available and accessible. The methodology applied is widely accepted, and the procedures followed are clearly described and intelligible. The statistical analyses have also been conducted appropriately and in an acceptable manner.

**********

what does this mean?). If published, this will include your full peer review and any attached files.

**Do you want your identity to be public for this peer review?**  If you choose “no”, your identity will remain anonymous but your review may still be made public.

For information about this choice, including consent withdrawal, please see our Privacy Policy

Reviewer #1: No

Reviewer #2: **Yes:** Daniel Maina Nderitu

**********

**Figure resubmission:**

**Reproducibility:**

To enhance the reproducibility of your results, we recommend that authors of applicable studies deposit laboratory protocols in protocols.io, where a protocol can be assigned its own identifier (DOI) such that it can be cited independently in the future. Additionally, PLOS ONE offers an option to publish peer-reviewed clinical study protocols. Read more information on sharing protocols at https://plos.org/protocols?utm_medium=editorial-email&utm_source=authorletters&utm_campaign=protocols

Attachment Submitted filename: Response to the Editor.docx

# Author comment: Author response to Decision Letter 2

**DOI:** 10.1371/journal.pdig.0001152.r004

13 Jul 2026

Attachment Submitted filename: Response_to_Reviewers_auresp_2.pdf

# Editor report: Decision Letter 2

**DOI:** 10.1371/journal.pdig.0001152.r005

**Authors:** Kezhi Li

17 Jul 2026

Socioeconomic Determinants of Malaria in Ugandan Children: An Interpretable Machine Learning Approach for  Public Health Policy

PDIG-D-25-01107R2

Dear Dr Mannan,

We are pleased to inform you that your manuscript 'Socioeconomic Determinants of Malaria in Ugandan Children: An Interpretable Machine Learning Approach for  Public Health Policy' has been provisionally accepted for publication in PLOS Digital Health.

Before your manuscript can be formally accepted you will need to complete some formatting changes, which you will receive in a follow-up email from a member of our team.

Please note that your manuscript will not be scheduled for publication until you have made the required changes, so a swift response is appreciated.

IMPORTANT: The editorial review process is now complete. PLOS will only permit corrections to spelling, formatting or significant scientific errors from this point onwards. Requests for major changes, or any which affect the scientific understanding of your work, will cause delays to the publication date of your manuscript.

If your institution or institutions have a press office, please notify them about your upcoming paper to help maximize its impact. If they'll be preparing press materials, please inform our press team as soon as possible -- no later than 48 hours after receiving the formal acceptance. Your manuscript will remain under strict press embargo until 2 pm Eastern Time on the date of publication. For more information, please contact digitalhealth@plos.org.

Thank you again for supporting Open Access publishing; we are looking forward to publishing your work in PLOS Digital Health.

Best regards,

Kezhi Li, PhD

Section Editor

PLOS Digital Health

***********************************************************

**Additional Editor Comments (if provided):**

**Reviewer Comments (if any, and for reference):**
